# Suppression of *spastin* Mutant Phenotypes by *Pak3* Loss Implicates a Role for Reactive Glia in AD-HSP

**DOI:** 10.3389/fnins.2020.00912

**Published:** 2020-09-04

**Authors:** Emily F. Ozdowski, Jill S. Wentzell, Stefanie M. Engert, Helena Abbott, Nina T. Sherwood

**Affiliations:** Department of Biology, Duke University, Durham, NC, United States

**Keywords:** Spastin, Pak3, Autosomal Dominant Hereditary Spastic Paraplegia, p21-activated kinase, subperineurial glia, microtubule severing proteins, reactive glia

## Abstract

Neurodegenerative mechanisms due to mutations in *spastin* currently center on neuronal defects, primarily in microtubule and endomembrane regulation. Spastin loss in *Drosophila* larvae compromises neuronal microtubule distribution, alters synaptic bouton morphology, and weakens synaptic transmission at glutamatergic neuromuscular junction (NMJ) synapses. Pak3, a p21-activated kinase that promotes actin polymerization and filopodial projections, is required for these *spastin* mutant defects; animals lacking both genes have normal NMJs. Here we show that Pak3 is expressed in central and peripheral glial populations, and reduction of Pak3 specifically in subperineurial glial cells is sufficient to suppress the phenotypes associated with *spastin* loss. Subperineurial glia in the periphery ensheathe motor neuron axons and have been shown to extend actin-based projections that regulate synaptic terminals during normal NMJ development. We find that these subperineurial glial projections are Pak3-dependent and nearly twice as frequent in *spastin* mutants, while in *Pak3, spastin* double mutants, neither glial projections nor synaptic defects are observed. Spastin deficiency thus increases Pak3-dependent subperineurial glia activity, which is in turn required for neuronal defects. Our results demonstrate a central role for Pak3-mediated, altered glial behavior in the neuronal defects due to *spastin* loss, and suggest that a similar reactive glia-mediated mechanism may underlie human AD-HSP pathogenesis.

## Introduction

The identification of human *SPAST* as the most common gene mutated in Autosomal-Dominant Hereditary Spastic Paraplegia two decades ago was a major advance, enabling multiple model systems to be leveraged toward understanding the requirement for Spastin in nervous system function (Hazan et al., 1999). Spastin likely serves to coordinately regulate the microtubule cytoskeleton and membrane components (reviewed in Lumb et al., 2012), and although clear consensus is still in progress, several working hypotheses have emerged for its neuronal roles. Potential processes include neural stem cell proliferation (Jeong et al., 2019), remodeling the endoplasmic reticulum and other endomembrane components (e.g., Park et al., 2010; Allison et al., 2019), lipid droplet function (Papadopoulos et al., 2015; Chang et al., 2019), axon regeneration (Stone et al., 2012), axon outgrowth (Wood et al., 2006), axon transport (Tarrade et al., 2006; Kasher et al., 2009), and synaptic morphology and transmission (Sherwood et al., 2004; Trotta et al., 2004).

*Drosophila spastin* was identified in a forward screen for genes involved in nervous system development (Sherwood et al., 2004). Just as *SPAST* mutations impair the longest axons of the central nervous system in humans, diminishing patient mobility, homozygous deletion of *Drosophila spastin* (the *spastin*^5.75^ allele) impairs mobility in adult flies, with the distal-most limbs also appearing the weakest. These adult flies are rarely viable, and although homozygous null larvae appear healthy, electrophysiological analysis of the larval neuromuscular junction (NMJ), a well-established model for vertebrate glutamatergic central synapses (Collins and DiAntonio, 2007), reveals reduced synaptic transmission due to defects at the presynaptic terminal (Sherwood et al., 2004; Ozdowski et al., 2011). This functional weakening is accompanied by distinctive changes in presynaptic arbor morphology. In contrast to wild type NMJs, *spastin*^5.75^ larvae have more highly branched axon terminal arbors, with smaller and more numerous synaptic boutons that often form bunched, rather than linear, arrays. Subcellularly, the normally continuous microtubule cytoskeleton appears sparse or undetectable in mutant terminal boutons, suggesting that Spastin’s microtubule severing activity is required for the generation and/or penetration of microtubules into distal sites. Consistent with this model, recent *in vitro* studies have shown that Spastin promotes stabilization and subsequent growth of severed microtubules, in addition to its well-established severing activity (Vemu et al., 2018; Kuo et al., 2019). These subcellular, morphological, and functional phenotypes in *spastin* mutants are all significantly rescued by low-level, neuron-specific expression of fly – or human – wild type Spastin, demonstrating that Spastin function is well-conserved between flies and humans, and is required within neurons (Sherwood et al., 2004; Du et al., 2010).

To understand the molecular and cellular mechanisms underlying the consequences of *spastin* loss, we executed a forward genetic screen for modifiers of *spastin* mutant phenotypes and identified *Pak3*, a member of the p21-activated serine/threonine kinase family (Ozdowski et al., 2011). Pak proteins are conserved from amoebae to humans, and typically activated downstream of Rac and Cdc42 small GTPase signaling, leading to alterations in the actin cytoskeleton and phosphorylation of a wide range of other protein targets (reviewed in Kumar et al., 2017; Semenova and Chernoff, 2017). The Paks are particularly well-studied because of their demonstrated roles inducing cell motility, invasion, and metastasis in several cancers, as well as contributing to cardiac and neurological disorders. Humans have six PAK genes (PAK 1–6), segregated into two structurally distinct groups. The group I PAKs, characterized by highly conserved autoinhibitory (AID) and kinase domains, form inactive dimers that depend upon Rac or Cdc42 binding for relief of autoinhibition and subsequent kinase activity. The group II PAKs lack an AID and do not require RhoGTPase binding for kinase activity. Of the proteins encoded by the three *Drosophila* Pak genes, *Pak*, *mushroom bodies tiny* (*mbt*), and *Pak3*, Pak is orthologous to the group I vertebrate Paks, mbt falls clearly into the group II subfamily, and Pak3, while less similar than Pak, is also considered a group I Pak due to its clear AID and ∼60% amino acid identity in the kinase domain (Mentzel and Raabe, 2005). Both overexpression and knockdown of *Drosophila Pak3* in various cell lines alters actin distribution and cell motility (Mentzel and Raabe, 2005; Asano et al., 2009; Ribeiro et al., 2014). *In vivo*, Pak3 is important in dorsal closure (Bahri et al., 2010), epidermal wound healing (Baek et al., 2012), podosome invasion during myoblast fusion (Duan et al., 2012), and border cell migration during oogenesis (Felix et al., 2015), all processes that require rearrangements of the actin cytoskeleton and changes in cell motility downstream of Rac signaling.

At the larval NMJ, ubiquitous, genetic loss of *Pak3* does not significantly affect synapse form or function; however, *Pak3* reduction in a *spastin* deficient background dramatically rescues the defects that characterize *spastin* mutants at this stage (Ozdowski et al., 2011). NMJ synapses in *Pak3*, *spastin* double mutants exhibit near-wild type morphology, with large and linearly-arrayed synaptic boutons. Subcellularly, continuity of the microtubule cytoskeleton within boutons is restored, and functionally, synaptic strength is indistinguishable from wild type levels. Absence of *Pak3* thus renders these neurons resistant to *spastin* loss.

Given this striking rescue, we sought to understand how the presence of Pak3 confers *spastin* mutant phenotypes. We examined the Pak3 expression pattern and used tissue-specific knockdown to determine the cells in which it acts when *spastin* is lost. Surprisingly, *Pak3* is not required in the motor neurons where Spastin functions, but rather, in the peripheral glia that ensheathe them. These glia provide essential support to the motor neurons, including extending filopodial projections that regulate the neuronal bouton arbor during development (Fuentes-Medel et al., 2009; Brink et al., 2012). We find that Pak3 promotes glial projections at the NMJ. Furthermore, in *spastin* mutants these glial projections are more numerous, while reduction of glial Pak3 suppresses both the projections and *spastin* mutant bouton morphology. Together, these data support a model in which *spastin* loss leads to increased Pak3-mediated glial activity, changing glial behavior such that they become toxic, rather than supportive, to synaptic terminals. Increasing evidence of a central role for reactive glial behavior in the progression of several major neurodegenerative diseases (Liddelow and Barres, 2017) makes these results particularly intriguing, as it suggests that pathogenesis in AD-HSP may be similarly mediated by reactive, toxic changes in a normally supportive subpopulation of glia.

## Materials and Methods

### *Drosophila* Stocks and Sources

Stocks and crosses were maintained on standard molasses-based food (Archon Scientific), except for experiments using the *flower* (*fwe*) loss of function alleles, *fwe^*DB*56^* and *fwe^*DB*25^* (gift of H. Bellen), which were raised on yeasted grape juice plates to facilitate recovery of the desired genotypes. All larvae and adults assayed were from crosses kept at 25°C. Unless otherwise specified, controls were *w*^1118^ flies, the common genetic background for transgenic strains; their NMJs are indistinguishable from wild type flies so are also referred to here as “wild type.” Driver lines were *repo-GAL4* (gift of M. Freeman), *gliotactin (gli)-GAL4* (gift of V. Auld), *e22c-Gal4 spaghetti-squash (sqh)-GAL4* (gift of D. Kiehart), *breathless* (*btl*)*-GAL4* (gift of M. Metzstein), and *elav^*C*155^-GAL4*, *Mef2-GAL4*, *Mhc-GAL4*, and *nSyb-GAL4* (all from the Bloomington Drosophila Stock Center, BDSC). *Pak3* alleles were *PBac{RB}Pak3^*e*00329^, P{XP}Pak3^*d*02472^* (abbreviated *Pak3*^*d*^ in Figures 3, 4; BDSC), *GAL4* insertion line *P{GawB}Pak3^*NP*4472^* (Kyoto Drosophila Genomics and Genetic Resources Center), and RNAi lines *Pak3^*NIG.*14895R^*^–2^ (National Institute of Genetics, NIG-FLY), *Pak3^*GL*00287^* (*P{TRiP.GL00287}attB*, BDSC), and *Pak3^*v*39844^* (P*{GD8481}v39844*, Vienna Drosophila Resource Center). Generation of the deletion allele *Df(3R)Pak3* (abbreviated *Pak3^*Df*^* in Figures 3, 4), was previously described (Ozdowski et al., 2011). *UAS-Pak3::GFP* constructs (gift of B. Baum) were injected into *Drosophila* embryos and two lines established: one insertion on chromosome II, *UAS-Pak3^50^::GFP*, and one on chromosome III, *UAS-Pak3^32^::GFP*. Tissue-specific expression of *UAS-Pak3^50^::GFP* rescues *Pak3* mutant adult lethality, consistent with the transgene producing functional Pak3 protein (E.F.O., unpublished results). *UAS-Pak3^32^::GFP* was used for *Pak3* overexpression in glia. Tissue-specific GFP was expressed using *UAS-mCD8::GFP* or *UAS-nls::GFP* flies (BDSC).

### Immunofluorescence

Wandering third-instar larvae were dissected in room temperature Phosphate Buffered Saline (PBS, Invitrogen) or HL3 Ringer’s medium without Ca^2+^ for < 20 min., or when quantifying glial projections, in HL3 Ringer’s containing 1.5 mM Ca^2+^ for <10 min. prior to fixation. Filets were then fixed in 4% paraformaldehyde (Electron Microscopy Sciences) for 30 min., washed in PBS with 0.2% Triton X-100 (PBST), and blocked in PBST with 5% normal goat serum, 0.01% bovine serum albumin, and 0.01% sodium azide for up to 2 h at room temperature. Filets were incubated overnight at 4°C in primary antibody diluted in block. Primary antibodies used were mouse polyclonal αPak3 (gift of S. Bahri, 1:250), rabbit αHRP (Jackson, 1:100), mouse αDLG (4F3 supernatant, Developmental Studies Hybridoma Bank [DSHB], 1:100), rat αElav (7E8A10, DSHB, 1:100), mouse αSlit (C555.6D, DSHB, 1:50), and mouse (mAb 3E6) or rabbit αGFP (Invitrogen, 1:300). Filets were then washed in PBST and incubated with the species-appropriate Alexa Fluor 488 or 568 secondary antibodies (Invitrogen) diluted 1:300 in block, for 2 h at room temperature or overnight at 4°C. After further washing, filets were mounted in Vectashield (Vector Laboratories) or ProLong Antifade (ThermoFisher).

### Synaptic Bouton and Glial Quantification and Imaging

Slide-mounted filets were scored with the experimenter blinded to larval genotypes, using an inverted fluorescence compound microscope (Zeiss Axiophot with a 63X, 1.2NA oil immersion objective or Zeiss Axio Imager with a 100X, 1.4NA oil immersion objective). Representative confocal images were obtained using a Zeiss 880 Airyscan confocal microscope and 63X, 1.2NA oil immersion or a 20X, 0.8NA dry objective. Images were false-colored magenta in Adobe Photoshop to aid visualization by color blind readers. Abdominal muscle 4 was identified for each hemisegment and type 1b synaptic bouton numbers were recorded. Type 1b bouton identity was confirmed by double staining with anti-DLG whenever possible (in all larvae for which anti-GFP immunostaining was not needed); otherwise, they were distinguished from type 1s boutons based on size and branch morphology. Terminal boutons, a measurement of synaptic arbor branching, were defined as any synaptic bouton with only one connection. This included all termini along the arbor and at the distal tips, regardless of size. Subperineurial glial projections were visualized using anti-GFP to visualize *gliotactin-GAL4*-driven expression of *UAS-mCD8::GFP*. Glial morphology at each main axon branch was characterized as (1) blunt, ending at synaptic boutons, (2) broad lamellipodia extending over synaptic boutons, (3) long, thin filopodia (gliopods) extending over synaptic boutons, away from the axon, or to neighboring axons, or (4) rounded structures (gliobulbs) that resemble boutons. Numbers of gliopods and gliobulbs were combined as a measure of glial projections. Glial and bouton counts from individual hemisegments were averaged for each larva and all values plotted as bee-swarm superplots using GraphPad Prism (Lord et al., 2020). Statistical significance of larval means was determined by two-tailed Student’s t-test in Microsoft Excel (Figure 2 and Supplementary Figure S1) or one-way ANOVA with Sidak’s *post hoc* test for multiple comparisons in GraphPad Prism (Figures 3, 4). P-values are denoted as ns for *p* > 0.05, ^∗^ for 0.01 < *p* ≤ 0.05, ^∗∗^ for 0.001 < *p* ≤ 0.01, and ^∗∗∗^ for *p* ≤ 0.001.

## Results

### Pak3 Is Expressed in Larval Glia

**FIGURE 1:**
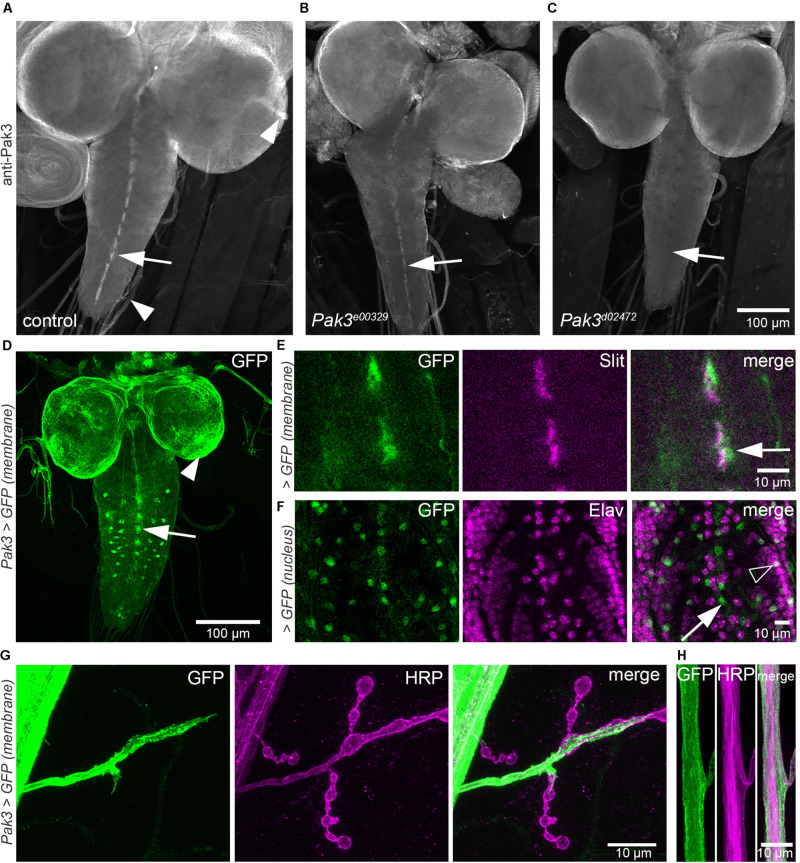
Pak3 is expressed in larval glia. **(A–C)** An anti-Pak3 polyclonal antibody reveals immunostaining consistent with glial cell expression: in larval ventral nerve cord (VNC) midline cells (**A**, arrow), along axonal projections emanating from the brain and VNC (**A**, arrowheads), and on the surface of the CNS. Expression is reduced in *Pak3^*e*00329^* mutants **(B)** and almost undetectable in *Pak3^*d*02472^* mutants **(C)**, consistent with the severity of RNA disruption in these alleles and thus supporting antibody specificity. **(D)** A second method of observing Pak3 expression using the *GAL4-UAS* reporter system, *Pak3-GAL4^ NP4472^* > *UAS-mcD8::GFP*, shows similar membrane GFP expression patterns (midline cells, arrow; surface cells, arrowhead), with additional cells visible in the VNC. **(E)** Midline membrane GFP expression (green) overlaps with anti-Slit (magenta; arrow), a marker for midline glia from embryonic through larval stages (Oliva et al., 2016). **(F)** Expression of nuclear-localized GFP (green) in animals with *Pak3-GAL4^ NP4472^* > *UAS-nls::GFP* confirms that the midline cells are anti-Elav-negative, indicating non-neuronal identity (arrow), but other GFP-positive cells in the VNC are neuronal (open arrowhead). **(G,H)** Along motor axons and at neuromuscular junction (NMJ) synapses, strong membrane GFP expression (green) extends beyond regions staining with the neuronal marker HRP (magenta), appearing to wrap the axons as well as project over proximal synaptic boutons, consistent with Pak3 expression in peripheral glia.

To begin to identify the specific cells in which *Pak3* loss prevents *spastin* mutant defects, we used a polyclonal antibody directed against the *Drosophila* Pak3 protein (kind gift of Sami Bahri; Bahri et al., 2010) to determine its endogenous expression pattern in larvae. Anti-Pak3 staining in wild type controls was strongly detected in the ventral nerve cord (VNC, analogous to the spinal cord of vertebrates) in a dashed pattern characteristic of midline glial cells (Figure 1A, arrow). Immunofluorescence was also seen in a thin layer on the surface of the larval brain, and along axonal projections emanating from the brain and ventral nerve cord (Figure 1A, arrowheads). The strength of the antibody signal correlated with predicted protein expression levels consistent with RNA disruption in different *Pak3* alleles, supporting the specificity of the antibody for Pak3 (compare Figures 1A–C).

Pak3 expression was also assessed using a *Pak3* promoter and GFP reporter system. The *Pak3* promoter-*GAL4* transgenic line *Pak3^*NP*4472^*, in which the GAL4 gene is inserted 160 bp upstream of the *Pak3* translational start site, was crossed to UAS-membrane GFP flies (*UAS-mCD8::GFP*; Lee and Luo, 1999). Larval progeny displayed strong GFP expression in cells that, similar to those revealed by the anti-Pak3 antibody, were arrayed in a dashed pattern characteristic of VNC midline glia (Figure 1D, arrow). Consistent with this identity, GFP-expressing cells at the midline colocalized with immunostaining for Slit, a protein secreted by midline glia (Figure 1E, arrow). They also failed to colocalize with anti-Elav, confirming their non-neuronal identity (Figure 1F, arrow). Also consistent with the anti-Pak3 antibody results, robust GFP signal was observed in glia covering the brain surface (Figure 1D, arrowhead) and ensheathing the motor axons between the ventral nerve cord and the periphery (Figures 1G,H). *Pak3^*NP*4472^*-driven GFP expression also revealed several other GFP-positive tissues that were not observed using the Pak3 polyclonal antibody, likely due to signal amplification conferred by both the GAL4-UAS system and immunostaining. These included a subset of Elav-positive neurons in the VNC (Figure 1F, open arrowhead), sensory neuron glia, and several tissues outside of the nervous system, particularly the trachea, oenocytes and salivary glands (data not shown). GFP expression was not detected in the larval body wall muscles, although Pak3 plays a significant role in myoblast fusion during embryonic stages (Duan et al., 2012). The expression pattern of the *Pak3* promoter*-GAL4* line thus corroborated the Pak3 antibody staining results, revealing surface, midline, and peripheral glia as major sites of Pak3 expression at this stage, but also suggested Pak3 functions in other cell types including neurons and trachea.

### Glial Knockdown of Pak3 Rescues the *spastin* Mutant Phenotype

**FIGURE 2:**
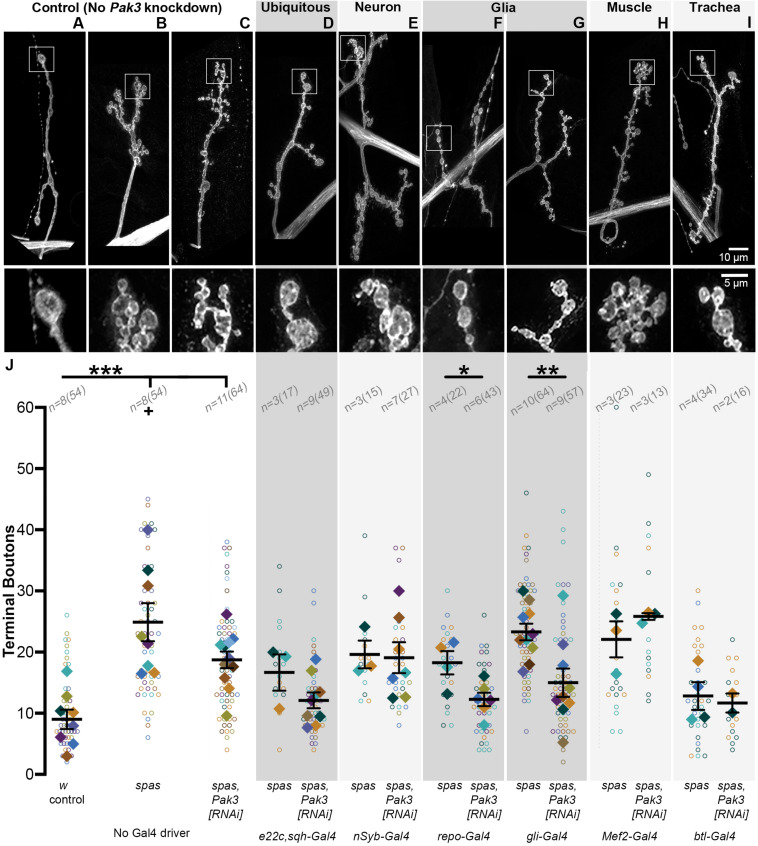
Glial, and specifically subperineurial glial, knockdown of *Pak3* rescues *spastin* synaptic defects. **(A–I)** Anti-HRP staining shows neuronal morphology at the NMJ. The area within the box is magnified below. **(A–C)** are genetic background controls in which *Pak3^*RNAi*^* is not expressed via GAL4 activation (**A** wild type; **B,C**: *spastin*^5.75^ mutant); **(D–I**) are representative arbors from *spastin*^5.75^ mutants in which *Pak3^*RNAi*^* is expressed in the specified tissues. **(A)** Wild type synaptic boutons are arrayed linearly with few branches, while **(B)** *spastin*^5.75^ loss of function mutants have highly branched arbors with many small, bunched boutons. **(C)** In the absence of a *GAL4* driver, the *Pak3^*RNAi*^* transgene has little effect on *spastin*^5.75^ morphology; terminal boutons remain significantly increased compared to wild type. **(D)** Ubiquitous expression of *Pak3^*RNAi*^* in the *spastin*^5.75^ background yields terminal boutons more similar in size, arrangement, and number to wild type. **(E)** *Pak3^*RNAi*^* expression in neurons does not rescue the *spastin* mutant phenotype, but **(F,G)** *Pak3* knockdown in all except midline glia with *repo-GAL4* **(F)**, or in subperineurial glia with *gliotactin-GAL4* **(G)**, significantly rescues *spastin*^5.75^. **(H,I)** Knockdown of *Pak3* in muscle **(H)** or trachea **(I)** show no difference in morphology from their *spastin*^5.75^ controls. **(J)** Synaptic morphologies are quantified by measuring the number of terminal boutons at each muscle 4 in a larva. In this and subsequent graphs, each open circle represents the quantity scored at an individual muscle; solid diamonds are the average of these counts for each larva, grouped by color; bars represent larval mean ± SEM; n(N) = number of larvae (number of muscles scored). “+” denotes two data points (y = 65 and 77) that fall outside of the *y*-axis range. The number of terminal boutons is statistically reduced compared to its driver-specific *spastin*^5.75^ control only when *Pak3^*RNAi*^* is expressed in glia or subperineurial glia. When compared to the *spastin*^5.75^ control, *Pak3^*RNAi*^* alleviates the *spastin* mutant phenotype if expressed ubiquitously, in all glia, or in subperineurial glia (*p* = 0.02). Statistical significance is calculated by Student’s *t*-test; asterisks signify *p* ≤ 0.001 (***), 0.001 < *p* ≤ 0.01 (**), or 0.01 < *p* ≤ 0.05 (*).

Given that loss of *Pak3* in the whole animal rescues the synaptic phenotype of *spastin* mutants, we next narrowed *Pak3* knockdown to specific tissues and looked for the same rescuing effect. Using tissue-specific GAL4 expression to drive RNAi targeting *Pak3* in a *spastin* null background (*spastin*^5.75^; Sherwood et al., 2004), we assayed type Ib glutamatergic synaptic boutons on muscle 4 of each larval hemisegment, as the neuronal arbors on this muscle are relatively simple, easily observed, and representative of the NMJ arbors across body wall muscles. Bouton and branching frequencies, measured primarily by terminal bouton number, were quantified for each genotype (Figure 2); increases in both at the larval NMJ are *spastin* loss of function hallmarks. *spastin*^5.75^ mutants had typically small and clustered boutons, averaging over twice as many terminal boutons as compared to *w*^1118^ controls (Figures 2A,B,J; *p* = 4.6 × 10^–4^). Separate experiments in which the total number of boutons was scored instead of just the terminals yielded equivalent results (Supplementary Figure S1, *w*^1118^ vs. *spas*; *p* = 1.7 × 10^–4^). To identify an effective *Pak3* RNAi line, we used *e22c-GAL4*, *spaghetti squash-GAL4* (*e22c,sqh-GAL4*; Franke et al., 2005) to drive ubiquitous expression of candidate RNAi transgenes in the *spastin*^5.75^ background, with the expectation that effective *Pak3* knockdown should recapitulate the suppression of *spastin* mutant phenotypes observed with genetic loss of *Pak3*. Of the publicly available lines we tested, *Pak3^*NIG.*14895R–2^* (henceforth referred to as “*Pak3^*RNAi*^*” *or “Pak3[RNAi]*”) was the most effective. In the absence of a GAL4 driver it did not significantly affect the *spastin* mutation (Figures 2C,J; *p* = 2.3 × 10^–4^ compared to controls; Supplementary Figure 1, *Pak3^*RNAi*^* vs. *Pak3^*RNAi*^, spas*; *p* = 1.3 × 10^–3^). When ubiquitously expressed, however, *Pak3^*RNAi*^* in the *spastin*^5.75^ null background resulted in larger and more linearly arrayed (less branched) boutons resembling wild type rather than *spastin*^5.75^ mutants (compare Figure 2D to the wild type control in Figure 2A versus *spastin*^5.75^ in Figures 2B,C). Terminal bouton number in *spastin*^5.75^ mutants also decreased to wild type levels, although due to difficulties obtaining healthy *e22c,sqh; spas* paired controls (i.e., animals with the *e22c,sqh-GAL4* driver and *spastin* mutation but no *Pak3^*RNAi*^*), this change did not reach statistical significance (Figure 2J; *p* = 0.13 for *e22c,sqh; spas* vs. *e22c,sqh* > *Pak3^*RNAi*^, spas*). However, comparison of *e22c,sqh* > *Pak3^*RNAi*^, spas* larvae to *e22c,sqh* > *Pak3^*RNAi*^* controls (ubiquitously expressing *Pak3^*RNAi*^* in a wild type, rather than *spastin* mutant background) showed no difference in bouton number, providing further support that ubiquitous expression of this *Pak3^*RNAi*^* transgene potently suppresses the effects of *spastin* mutation (Supplementary Figure S1, *p* = 0.67).

We then compared the consequence of *Pak3* reduction using the pan-neuronal driver *nSyb-GAL4* (Jenett et al., 2012), the glial driver *repo-GAL4* (Sepp et al., 2001), the muscle drivers *Mef2-GAL4* and *Mhc-GAL4*, and the tracheal driver *btl-GAL4* (Shiga et al., 1996), as all of these cell types have been implicated in regulating *Drosophila* NMJ synapses and show evidence of Pak3 expression as discussed above. *nSyb-GAL4-*, *Mef2-GAL4*-, and *Mhc-GAL4*-mediated *Pak3^*RNAi*^* expression all failed to alleviate *spastin*^5.75^, excluding neurons (*p* = 0.90) and muscles (*p* = 0.28 and 0.79 for *Mef2* and *Mhc*, respectively), as sites of Pak3’s deleterious effects (Figures 2E,H,J; *Mhc-GAL4* data not shown, *n* = 3 and 4 larvae for control and experimental groups). *Spastin* mutant synapses were rescued only with glial-specific reduction of *Pak3* using *repo-GAL4*, which drives expression in all glia except for those at the midline (Figures 2F,J; *p* = 1.9 × 10^–3^ for *repo-GAL4, spas* vs. *repo-GAL4* > *Pak3^*RNAi*^, spas*). *Repo-GAL4*-mediated *Pak3* knockdown restored synapse morphology of *spastin*^5.75^ mutants to wild type (Figure 2F), comparable to ubiquitous (*e22c,sqh-GAL4*-driven) knockdown (Figure 2D) or genetic loss of *Pak3* (Ozdowski et al., 2011). Parallel experiments showed no significant difference in bouton number between glial *Pak3* knockdown in wild type versus *spastin* mutant backgrounds, further supporting a requirement for glial Pak3 in the *spastin* mutant phenotype (Supplementary Figure S1, *p* = 0.07). Simultaneous expression of two independently generated *Pak3* RNAi transgenes (*Pak3^*GL*0028^* and *Pak3^*v*39844^*) with *repo-GAL4* also suppressed the *spastin*^5.75^ phenotype (Supplementary Figure S2, *Pak3^*GL*0028^*,*Pak3^*v*39844^*, *spas* controls vs. *repo-GAL4* > *Pak3^*GL*0028^*,*Pak3^*v*39844^*, *spas*; p = 1.3 × 10^–3^), and had no effect when expressed using the neuron-specific *elav-GAL4* driver (Supplementary Figure S2, *Pak3^*GL*0028^*,*Pak3^*v*39844^*, *UAS-Dcr2, spas* controls vs. *elav^*C*155^-GAL4* > *Pak3^*GL*0028^*,*Pak3^*v*39844^*, *UAS-Dcr2, spas*; *p* = 0.32), supporting that these results are specific to Pak3 reduction and not due to RNAi off-target interactions. We were unable to draw any conclusions regarding a role for trachea using this tissue-specific knockdown approach since the *btl-GAL4* transgene alone with *spastin*^5.75^ had near-wild type morphology (Figures 2I,J). Regardless, together with the Pak3 expression pattern, these data substantiate glia as the major cell type in which Pak3 acts to cause neuronal defects when *spastin* is lost.

### Pak3 Acts in the Subperineurial Glia

The *Drosophila* nervous system includes multiple glial cell types, each with distinct functions that bear striking molecular and behavioral parallels to vertebrate glial subtypes (reviewed in Freeman, 2015; Yildirim et al., 2019). Motor axons projecting from the larval VNC to the NMJ are ensheathed by three layers of glia. The outermost, perineurial glial (PG) layer, and middle, subperineurial glia (SPG) layer, effectively shield the axons from their surroundings, just as they do in the CNS where, as the “surface glia” (at least some of which express Pak3, see Figure 1), they form the blood-brain-barrier. The innermost layer, the wrapping glia, directly contact individual axons and mediate their homeostasis. All three layers ensheathe the motor axons along their lengths, typically ending at or near the first synaptic bouton at the NMJ. Beyond this point, the perineurial and subperineurial, but not wrapping, glial layers extend dynamic, actin-based processes that interact with synaptic arbors at the NMJ (Fuentes-Medel et al., 2009; Brink et al., 2012). We found that expression of *Pak3^*RNAi*^* using the subperineurial-specific glia driver *gliotactin (gli)-GAL4* (Sepp et al., 2000) rescued the *spastin* mutant phenotype as effectively as *repo-GAL4* or *e22c,sqh-GAL4*, reducing terminal bouton number by 35% and restoring wild type morphology (Figures 2G,J; *p* = 5.7 × 10^–3^ for *gli-GAL4, spas* vs. *gli-GAL4* > *Pak3^*RNAi*^, spas*). Subperineurial glia can therefore account for the majority of *spastin*^5.75^ rescue conferred by *Pak3* loss, supporting the idea that neuronal damage due to *spastin* loss requires Pak3 in the SPG.

### SPG Projections Depend on Pak3 and Are Enhanced in *spastin* Mutants

**FIGURE 3:**
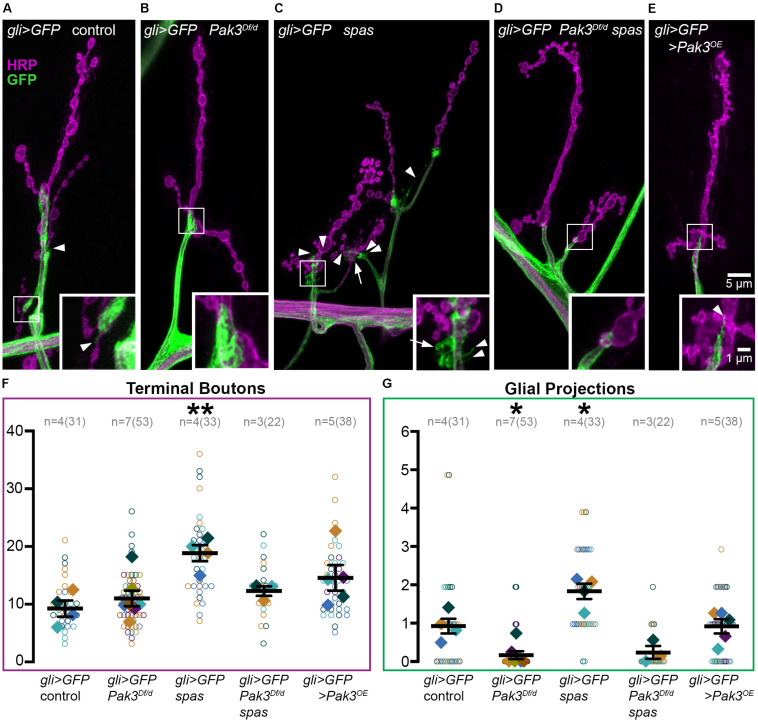
Subperineurial glia projections depend on Pak3 and are doubled in *spastin* mutants. **(A–E)** Anti-HRP shows neuronal morphology in magenta, anti-GFP shows glial morphology in green. **(A)** *gli-GAL4, UAS-mCD8::GFP* (*gli* > *GFP*) controls have linearly arrayed synaptic boutons and average one glial projection (arrowhead) per muscle 4. **(B)** *Pak3* loss of function mutants, *gli* > *GFP*; *Pak3^*Df*^*/*Pak3^*d*02472^* (abbreviated “*Pak3^*Df/d*^*”) have similarly arrayed bouton arbors but very few glial projections. **(C)** *spastin*^5.75^ loss of function, *gli* > *GFP*; *spas*, causes extra glial projections in addition to extra terminal boutons. These projections include thin glial filopodia (arrowheads) and round glial projections (arrows). **(D)** Consistent with these extra glial projections requiring Pak3 function, *gli* > *GFP*; *Pak3^*Df*^, spas*/*Pak3^*d*02472^, spas* double mutants display few glial projections, and have wild type bouton arbors. **(E)** Overexpression of *Pak3* in subperineurial glia does not affect glial projections or terminal bouton numbers compared to controls, although small, clustered terminal boutons are observed. **(F,G)** Graphs of individual (open circles) and averaged (solid diamonds) values per larva for terminal boutons **(F)** and glial projections **(G)** are shown for each of the genotypes in **(A–E)**. Significant differences relative to gli > GFP controls are calculated by one-way ANOVA followed by Sidak’s multiple comparisons test. *P*-values are denoted as * for 0.01 < *p* ≤ 0.05, and ** for 0.001 < *p* ≤ 0.01.

How might Pak3 in subperineurial glia damage neuronal synapses? One possibility is that deleterious effects are mediated by the SPG projections that interact with the synaptic arbor. Given that Pak3 promotes actin polymerization and that these glial projections are actin-rich (Brink et al., 2012), we predicted that neurotoxic Pak3 in *spastin* mutants would be reflected in enhanced numbers of these glial projections. We used *gli-GAL4* driven-expression of *UAS-mCD8::GFP* to visualize SPG membranes (Figures 3A–D, green) and anti-HRP immunostaining to label neurons (magenta) in (Figure 3A) wild type, (Figure 3B) *Pak3^*Df*^/Pak3^*d*02472^* mutant, (Figure 3C) *spastin*^5.75^ mutant, or (Figure 3D) the *Pak3^*Df*^/Pak3^*d*02472^, spastin^5.75^* double mutant backgrounds. These animals were dissected in physiological levels of Ca^2+^ since glial projections are highly Ca^2+^-sensitive (Brink et al., 2012). Gliopods (long, thin projections) and gliobulbs (rounded, resembling a bouton) were scored separately, but no genotype-dependent changes were observed between the two morphologies so they were combined. In the control background (“*gli* > *GFP* control” in Figure 3A), we observed on average 10 terminal boutons (Figure 3F) and a single SPG projection (Figure 3G) per muscle 4. Glial projections in *Pak3^*Df*^/Pak3^*d*02472^* loss of function animals were rarer, averaging only one projection for every 5 muscles scored (Figures 3B,G; *p* = 0.017 compared to controls). This demonstrated that Pak3 is generally required for the formation of SPG projections (Figure 3G), but that these projections are not required for normal synaptic bouton arbors, which were no different from controls (Figure 3F, *p* = 0.97). In contrast, *spastin*^5.75^ mutants had twice as many projections compared to controls (Figures 3C,G; *p* = 0.011), consistent with increased Pak3-mediated glial activity when *spastin* is lost, and correlating with the increase in average terminal bouton numbers (Figures 3C,F; *p* = 8.0 × 10^–3^). Animals deficient for both *spastin* and *Pak3* had near-wild type bouton numbers as expected (Figures 3D,F; *p* = 0.87), and similar to the *Pak3* mutant alone, glial projections were virtually absent (Figures 3D,G; *p* > 0.99). Thus, SPG projections depend on Pak3, are enhanced in *spastin* mutants, which have aberrant synaptic arbors, and are sparse in *Pak3, spastin* double mutants, which have normal synaptic arbors.

Given that Pak3 in SPG is necessary for *spastin* mutant synapses, we asked whether overexpression of *Pak3* in wild type SPG is sufficient to elicit synaptic defects characteristic of *spastin* loss. *Pak3* was overexpressed in GFP-labeled SPG using *gli-GAL4* to drive expression of *UAS-Pak3* and *UAS-mCD8::GFP* in an otherwise wild type background. *UAS-Pak3* induces actin-rich projections in the synaptic bouton arbor when overexpressed in neurons (Ozdowski et al., 2011) and rescues adult lethality in *Pak3* loss of function animals (E.F.O. unpublished results). *Pak3* overexpression in SPG induced small boutons like those seen in *spastin* mutants (Figure 3E), but did not significantly increase terminal bouton number (Figure 3F, *p* = 0.21 compared to controls) or alter glial projections (Figures 3E,G; *p* > 0.99). Closer examination of the relationship between terminal bouton and glial projection numbers at individual muscles confirmed an absence of synapse-specific correlation between neuronal boutons and glial projections in wild type as well as *spastin* loss of function animals (Pearson’s correlation coefficient r_*wildtype*_ = −0.06 and r_*spastin*_ = −0.2), despite their strong association on average. While this could suggest that SPG projections are simply an epiphenomenon, their intimate association with the NMJ and role in sculpting synaptic arbors (Fuentes-Medel et al., 2009) argue for their functional relevance in this context.

### Pak3-Mediated Toxicity Shows Specificity to *spastin* Loss

**FIGURE 4:**
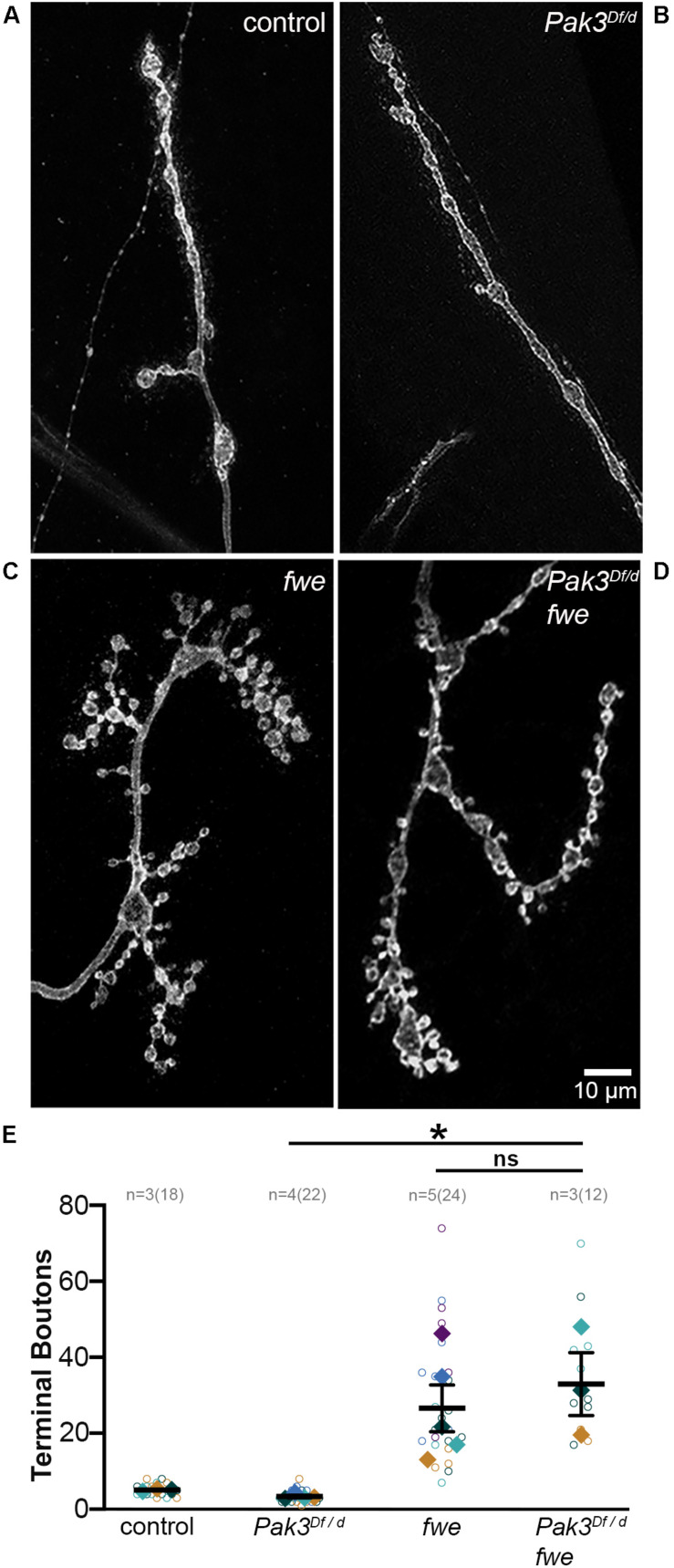
*Pak3* loss of function does not rescue all supernumerary bouton mutants. **(A–D)** Anti-HRP staining shows neuronal morphology at the NMJ. **(A)** Wild type synaptic boutons are arrayed linearly with only a few branches and **(B)** *Pak3* loss of function mutants, *Pak3^*Df*^*/*Pak3^*d*02472^*, show no differences from wild type. **(C)** Loss of function mutations in *flower* (*fwe^*DB*56^*/*fwe^*DB*25^*) result in extreme branching of the neuronal arbor and small bouton size, similar to *spastin* mutant morphology. **(D)** Loss of *Pak3* function does not rescue the supernumerary bouton phenotype in *fwe* mutants, indicating that *Pak3* loss is not a generalized mechanism to reduce extra synaptic branching. **(E)** Individual terminal bouton numbers (open circles) and larval averages (solid diamonds) are shown for each of the genotypes in **(A–D)**. Significant differences are calculated by one-way ANOVA and Sidak’s multiple comparisons test. * denotes 0.01 < *p* ≤ 0.05.

The striking efficacy of *Pak3* removal in suppressing defects due to *spastin* loss suggested that Pak3-mediated changes in glial behavior might be a common mechanism underlying neuronal dysfunction. To test this possibility, we investigated genetic mutants in *flower* (*fwe*), which like *spastin* mutants, exhibit small, highly branched, supernumerary boutons (Figure 4; Yao et al., 2009, 2017). Mutants bearing these morphological hallmarks are thought to be functionally related, most likely in aspects of endosomal cycling (Dickman et al., 2006), and we reasoned that functionally related proteins are more likely to have conserved regulatory mechanisms. We compared the NMJs of trans-heterozygous loss of function mutants in *fwe* (*fwe^*DB*56^/fwe^*DB*25^*) with and without *Pak3* loss of function, and found no rescue of terminal bouton number or overall morphology of *fwe* mutants with *Pak3* loss (compare Figures 4C,D; *p* = 0.96). Loss of *Pak3* in SPG thus does not act as a non-specific suppressor of synaptic defects; rather, its rescuing role is specific, at least in part, to the sequelae of *spastin* loss.

## Discussion

Through an unbiased, forward genetic screen for modifiers of Spastin activity, we previously showed that the actin regulator Pak3 is required for the manifestation of morphological and functional synaptic defects due to *spastin* loss (Ozdowski et al., 2011). Here, we have determined that Pak3 functions in larval subperineurial glia cells, where it promotes projections that extend into the NMJ synaptic arbor. The SPG projections are highly dependent on Pak3, whether in wild type animals, where these glia serve supportive roles in synaptic development (e.g., Fuentes-Medel et al., 2009, 2012), or in *spastin* mutants, where the projections are twice as frequent and synapses are defective.

The ability of *Pak3* loss to suppress *spastin* mutant defects suggests that Pak3-dependent glial projections are a critical aspect of SPG toxicity when *spastin* is lost. Excess glial projections could, for example, reflect phagocytic behavior gone awry, given that wild type projections positively regulate synaptic arbor morphology through this mechanism (Fuentes-Medel et al., 2009). Indeed, these phagocytic engulfment events, mediated by the Draper receptor, have been shown to play both neuroprotective and neurodegenerative roles in the CNS, depending on context (reviewed in Logan, 2017; Hilu-Dadia and Kurant, 2019). Whether SPG projections in *spastin* mutants have analogous functions remains to be determined, however, particularly given the lack of correlation we observed between synaptic bouton and SPG morphologies at individual NMJs in both wild type and mutant animals. Even with rapid dissection, it is possible that our fixed preparations fail to accurately capture the distribution of these dynamic glial structures and their precise relationship to associated synaptic boutons. Additionally, glial projections may represent only a subset of Pak3-mediated changes within SPG cells contributing to the *spastin* mutant phenotype. Finally, while Pak3 in SPG is required for the *spastin* mutant phenotype, other cell types may also be involved. We do not yet know whether *spastin* loss affects perineurial glia behavior, for example, or the underlying muscle or adjacent trachea, all of which have been shown to interact with the presynaptic arbor (Fuentes-Medel et al., 2009; Brink et al., 2012; Chen et al., 2019).

In addition to defining the contribution of these additional cell types, future experiments will be important to determine whether Pak3 is altered in response to *spastin* loss within the same SPG cells, or if changes in Pak3 are induced cell non-autonomously. Our earlier results used tissue-specific rescue to demonstrate that *spastin* is primarily required in neurons (Sherwood et al., 2004; Du et al., 2010), and expression studies in mouse and human also support this neuron-specific requirement (Ma et al., 2006). If so, this argues for a model in which *spastin*-deficient neurons send a signal that leads to Pak3 upregulation in SPG, and subsequent deleterious effects on synaptic boutons. Given that our neuron-specific rescue of the *spastin* phenotype was incomplete, however, we cannot rule out roles for Spastin in other cells (Sherwood et al., 2004). *Spastin* RNAi has been ineffective in our hands, and neither antibodies nor promoter-*GAL4* lines have convincingly revealed its endogenous expression pattern in *Drosophila*. However, Spastin expression has been shown via Western blots in normal human primary cerebellar astrocytes, glioblastoma cells, and rodent reactive astrocytes, consistent with possible roles in non-neuronal cell types and specifically glia (Ma et al., 2006; Draberova et al., 2011; Havlicek et al., 2014).

Although they remain the “support cells” of the nervous system, the idea that glia can also induce neuronal toxicity, particularly in the context of other defects, is increasingly evident. In flies, the requirement for Pak3 to elicit neuronal dysfunction during *spastin* loss bears striking parallels to the glial-derived, prodegenerative signaling molecule Eiger (the mammalian TNF-α ortholog; Keller et al., 2011). Neither protein is required for normal synapse development; rather, it is their glial deployment in mutant contexts (Pak3 in *spastin* mutants, Eiger in *ankyrin* mutants), that promotes neuronal dysfunction. Our inability to generate excess glial projections or significantly alter bouton number by overexpressing *Pak3* in wild type animals supports, additionally, that activation of these glial pathways is only damaging to neurons in specific mutant contexts. Conversely, as seen with the *fwe* experiment, absence of these proteins does not serve as a generalized mechanism by which even potentially related synaptic defects can be rescued.

Dramatic changes in glial morphology and behavior, from non-reactive to reactive states, are also observed in mammalian nervous systems in response to damage (reviewed in Liddelow and Barres, 2017). Ischemic versus inflammatory insults induce distinct forms of reactive astrocytes; while ischemic damage leads to “A2” reactive astrocytes that support repair and survival, neuroinflammatory insults induce “A1” reactive astrocytes that are toxic to synapses. These A1 reactive astrocytes are now thought to play causal roles in the progression of several neurodegenerative diseases including ALS, Alzheimer’s, Parkinson’s, and Huntington’s. Our results suggest that AD-HSP is also a candidate for this list, and that putative reactive glial behavior could be mediated by excess Pak activity. *Drosophila* Pak3 bears considerable amino acid identity and functional correspondence to the vertebrate group 1 Paks (Mentzel and Raabe, 2005), which are extensively studied for their roles in several cancers and actively targeted in drug development (reviewed in Rane and Minden, 2019). Should evidence for a Pak-mediated reactive glial mechanism be revealed in AD-HSP, the group I Paks will be promising targets for therapeutic intervention.

## Data Availability Statement

The raw data supporting the conclusions of this article will be made available by the authors, without undue reservation, to any qualified researcher.

## Author Contributions

EO and NS contributed to the conception and experimental design. JW, SE, and HA generated and characterized genetic tools, including the efficacy of GAL4 drivers and RNAi lines, and obtained pilot data on bouton and glial phenotypes. EO performed dissections and immunofluorescence with anti-Pak3, -Slit, -Elav, and -GFP, and quantified adult eclosion rates, boutons for the *fwe* mutant experiment, and glial projections. EO, NS, and HA performed genetic crosses, larval dissections, immunofluorescence, acquired confocal images, and performed statistics on numeric data for the tissue-specific RNAi experiments. NS wrote the first draft of the manuscript. EO compiled the figures and figure legends and contributed to manuscript revision. All authors read and approved the written work.

## Conflict of Interest

The authors declare that the research was conducted in the absence of any commercial or financial relationships that could be construed as a potential conflict of interest.

## Supplementary Material

FIGURE S1 Ubiquitous or glial-specific *Pak3^*R**NAi*^* expression in wild type versus *spastin* mutant backgrounds suppresses the effects of *spastin* mutation. From left to right: Loss of *spastin* in Controls induces a nearly twofold increase in total bouton number (left two genotypes), and this effect is not altered by the presence of the *Pak3^*R**NAi*^* transgene (next two genotypes). Ubiquitous *Pak3^*R**NAi*^* expression makes larval synapses resistant to *spastin* removal, as bouton numbers in wild type (left; *e,sqh* > *Pak3[RNAi]*) and *spastin* mutant (right; *e,sqh* > *Pak3[RNAi],spas*) backgrounds are not significantly different. Neither neuron- nor muscle-specific *Pak3^*R**NAi*^* expression suppress supernumerary boutons in *spastin* mutants. These results are consistent with those of Figure 2. The effects of tracheal *Pak3^*R**NAi*^* expression are inconclusive, as the *btl-GAL4* driver alone may suppress the *spastin* mutant phenotype independent of *Pak3* (see Figure 2). Statistical significance is determined by Student’s *t*-test. *P*-values are denoted as ns for *p* > 0.05, * for 0.01 < *p* ≤ 0.05, ** for 0.001 < *p* ≤ 0.01, and *** for *p* ≤ 0.001.

FIGURE S2 Expression of two different *Pak3* RNAi transgenes, and use of a different pan-neuronal driver, independently support alleviation the *spastin* mutant phenotype by glial, and not neuronal, *Pak3* knockdown. Simultaneous pan-neuronal expression of the *Pak3^*G**L0028*^* and *Pak3^*v*39844^* RNAi transgenes (“*Pak3[2xRNAi]*”) does not alleviate supernumerary boutons of *spastin* mutants (*p* = 0.32 compared to paired control). The neuronal driver used in these experiments is *elav^*C155*^-GAL4* rather than *nsyb-GAL4*; *Dcr2* was also expressed to increase RNAi efficacy. In contrast, pan-glial expression of these *Pak3* RNAi transgenes has the same effect as *Pak3^*N**IG.1489*5R^*^–2^ RNAi expression (from Figure 2 and Supplementary Figure S1), reducing terminal bouton number in *spastin* mutants to wild type levels (*p* = 1.3 × 10^–3^; Student’s *t*-test).
