# Supplementary figures and images for: Suppression of spastin Mutant Phenotypes by Pak3 Loss Implicates a Role for Reactive Glia in AD-HSP
Source: Front Neurosci. 2020 Sep 4;14:912. doi: 10.3389/fnins.2020.00912 (PMC7499821; doi:10.3389/fnins.2020.00912)

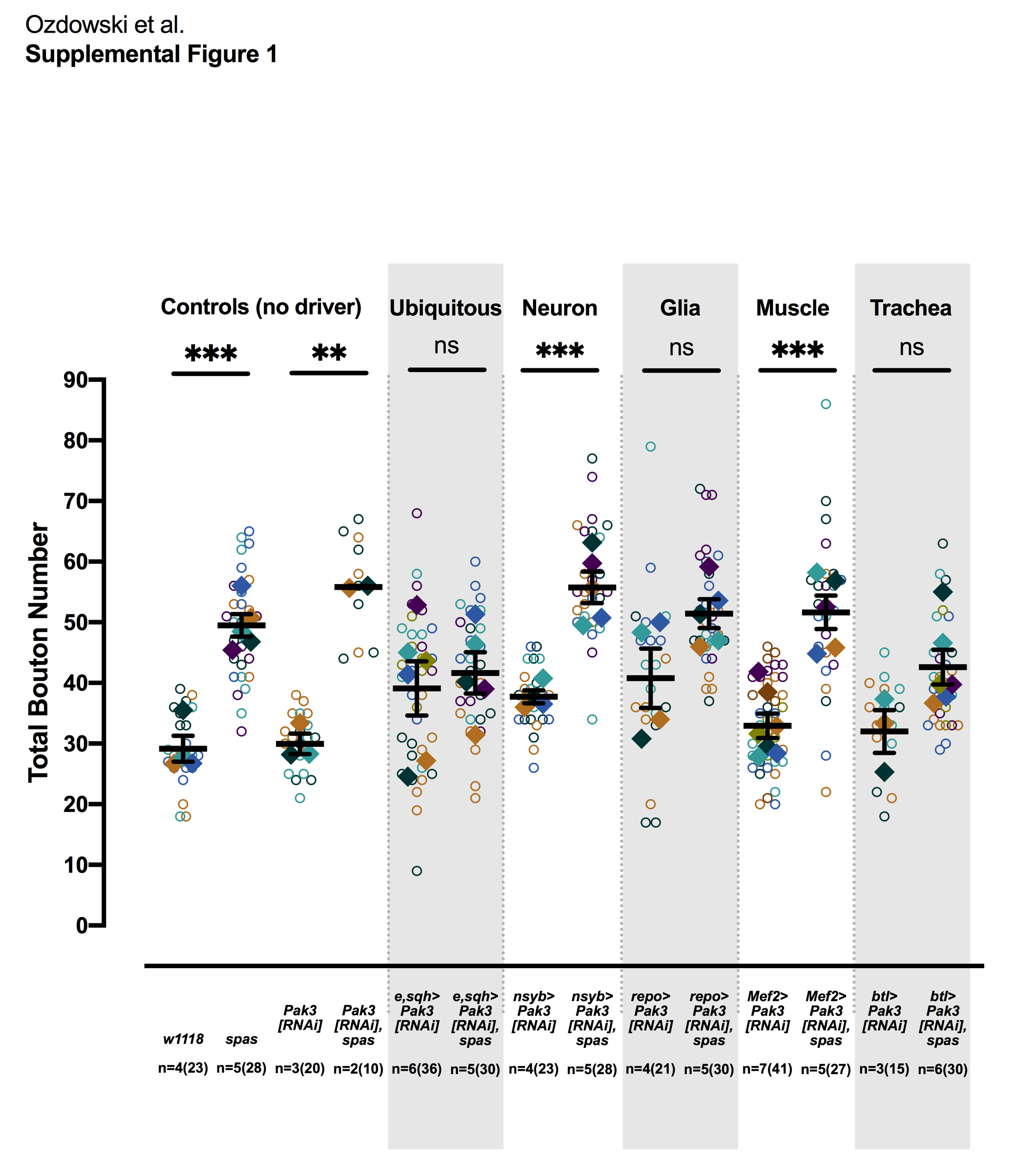

Supplement: FIGURE S1 — Ubiquitous or glial-specific Pak3RNAi expression in wild type versus spastin mutant backgrounds suppresses the effects of spastin mutation. From left to right: Loss of spastin in Controls induces a nearly twofold increase in total bouton number (left two genotypes), and this effect is not altered by the presence of the Pak3RNAi transgene (next two genotypes). Ubiquitous Pak3RNAi expression makes larval synapses resistant to spastin removal, as bouton numbers in wild type (left; e,sqh > Pak3[RNAi]) and spastin mutant (right; e,sqh > Pak3[RNAi],spas) backgrounds are not significantly different. Neither neuron- nor muscle-specific Pak3RNAi expression suppress supernumerary boutons in spastin mutants. These results are consistent with those of Figure 2. The effects of tracheal Pak3RNAi expression are inconclusive, as the btl-GAL4 driver alone may suppress the spastin mutant phenotype independent of Pak3 (see Figure 2). Statistical significance is determined by Student’s t-test. P-values are denoted as ns for p > 0.05, * for 0.01 < p ≤ 0.05, ** for 0.001 < p ≤ 0.01, and *** for p ≤ 0.001. [file Image_1.TIFF]

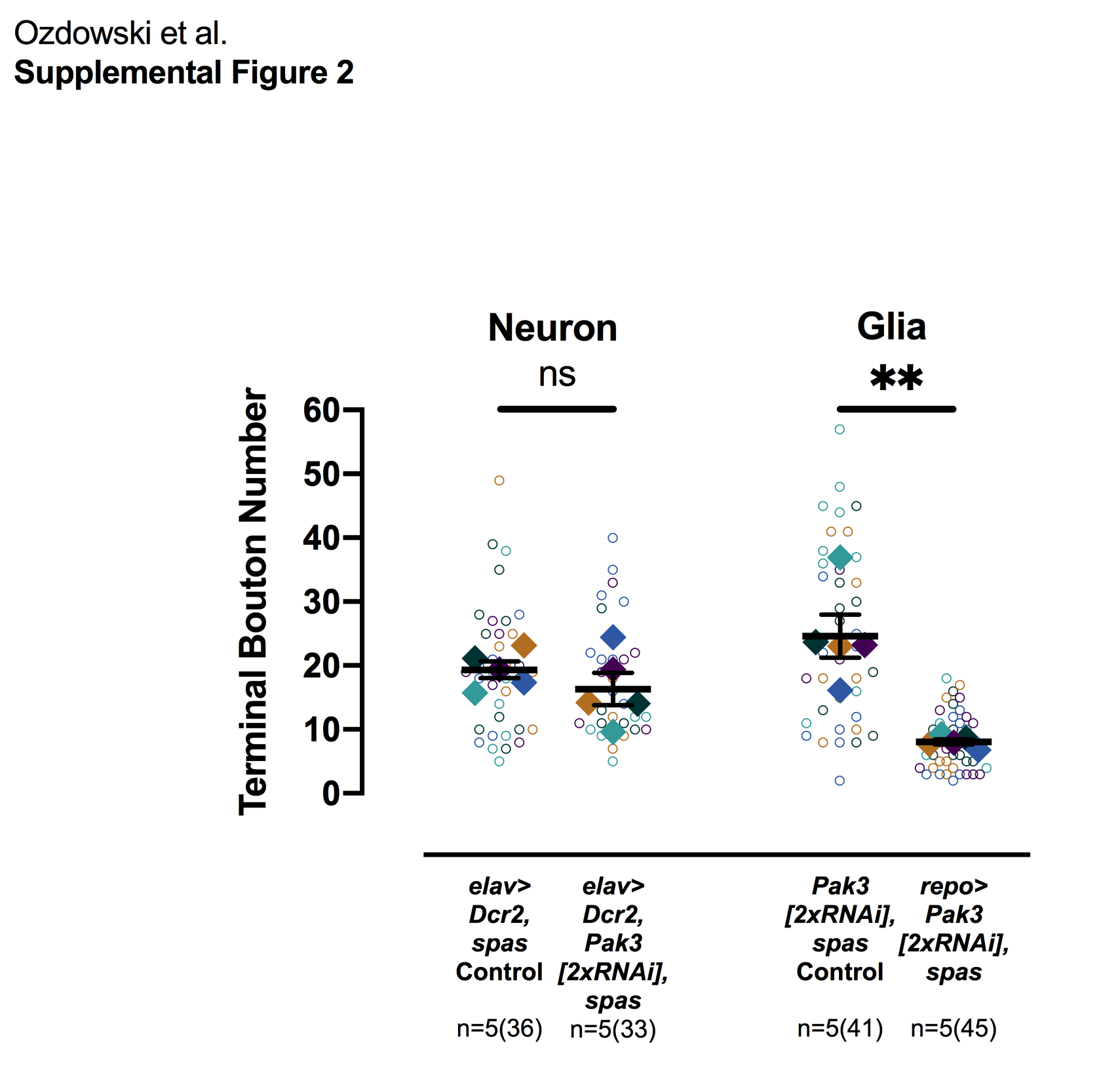

Supplement: FIGURE S2 — Expression of two different Pak3 RNAi transgenes, and use of a different pan-neuronal driver, independently support alleviation the spastin mutant phenotype by glial, and not neuronal, Pak3 knockdown. Simultaneous pan-neuronal expression of the Pak3GL0028 and Pak3v39844 RNAi transgenes (“Pak3[2xRNAi]”) does not alleviate supernumerary boutons of spastin mutants (p = 0.32 compared to paired control). The neuronal driver used in these experiments is elavC155-GAL4 rather than nsyb-GAL4; Dcr2 was also expressed to increase RNAi efficacy. In contrast, pan-glial expression of these Pak3 RNAi transgenes has the same effect as Pak3NIG.14895R–2 RNAi expression (from Figure 2 and Supplementary Figure S1), reducing terminal bouton number in spastin mutants to wild type levels (p = 1.3 × 10^–3^; Student’s t-test). [file Image_2.TIFF]
